# High Incidence of Gestational Trophoblastic Disease in a Third-Level University-Hospital, Italy: A Retrospective Cohort Study

**DOI:** 10.3389/fonc.2021.684700

**Published:** 2021-05-05

**Authors:** Giampiero Capobianco, Elettra Tinacci, Laura Saderi, Francesco Dessole, Marco Petrillo, Massimo Madonia, Giuseppe Virdis, Alessandro Olivari, Davide Adriano Santeufemia, Antonio Cossu, Salvatore Dessole, Giovanni Sotgiu, Pier Luigi Cherchi

**Affiliations:** ^1^ Gynecologic and Obstetric Clinic, Department of Medical, Surgical and Experimental Sciences, University of Sassari, Sassari, Italy; ^2^ Clinical Epidemiology and Medical Statistics Unit, Department of Medical, Surgical and Experimental Sciences, University of Sassari, Sassari, Italy; ^3^ Institute of Urology, University of Sassari, Sassari, Italy; ^4^ Oncologic Unit, ATS Sardegna, Ospedale Civile Alghero, Sassari, Italy; ^5^ Institute of Pathology, University of Sassari, Sassari, Italy

**Keywords:** gestational throphoblastic disease, serum human chorionic gonadotrophin, obstetric outcome, epidemiology, prognostic

## Abstract

**Introduction:**

to assess incidence, prognosis and obstetric outcome of patients treated for gestational trophoblastic disease GTD in a twenty-year period. Incidence, prognosis and obstetric outcome of gestational throphoblastic disease

**Methods:**

retrospective study.

**Results:**

Fifty-four cases of GTD: 46 (85.18%) cases of Hydatidiform mole (HM); 8 cases of Persistent Gestational Trophoblastic Neoplasia (GTN) (14.81%): 6/8 cases (75%) GTN not metastatic; 2/8 cases (25%) GTN metastatic. In both cases, the metastases occurred in the lungs. In 3 out of 8 GTN cases (37.5%) a histological picture of choriocarcinoma emerged. The incidence of GTD cases treated from 2000 to 2020 was 1.8 cases per 1000 deliveries and 1.3 cases per 1000 pregnancies. Of the 54 patients, 30 (55.56%) presented showed normal serum hCG levels without the need for chemotherapy. On the other hand, 24 patients (44.44%) developed a persistent trophoblastic disease and underwent adjuvant therapy. The negative prognostic factors that affected the risk of persistence of GTD were: serum hCG levels at diagnosis > 100,000 mUI/ml; characteristic “*snow storm*” finding at the ultrasound diagnosis; a slow regression of serum hCG levels during follow-up; the persistence of high serum hCG levels (especially if > 1000 mUI/ml one month after suction curettage) that was the main risk factor for resistance to first-line chemotherapy. There were 10 pregnancies in total following treatment. Patients’ survival in our study was 100%.

**Discussion:**

Although GTD is a rare disease, its incidence was 1.3 cases per 1,000 pregnancies in Sardinia, Italy, higher if compared with mean national and worldwide incidence.

## Introduction

Gestational trophoblastic disease (GTD) is a heterogeneous group of epithelial tumors which originate from placental trophoblastic tissue after abnormal fertilization and relate to a pregnancy event (i.e., abortion, extra-uterine or term/preterm pregnancy). Trophoblast shows limited histolytic, angiotropic, and invasive power, not exceeding thin myometrial basal decidua. Chorionic neoplasms, which are histologically similar to the chorionic villus, have invasive morphological and proliferative attitudes (1).

From a clinical-pathological point of view, we can distinguish the hydatidiform mole (HM) (complete and partial), which represents the most common form (80% of cases) and is a premalignant disease and the malignant gestational trophoblastic neoplasia (GTN), that may be nonmetastatic or metastatic (1, 2). The latter can include: Invasive mole (15% of cases); choriocarcinoma (a rare form that makes up about 5% of cases); placental site trophoblastic tumor (PSTT) (3), extremely rare; epithelioid trophoblastic tumor (ETT), even rarer (4, 5).

GTD burden can vary: in North America and Europe the incidence ranges from 0.57 to 1.1 cases per 1,000 pregnancies, whereas in Asia ~2.0 cases per 1,000 pregnancies (1). The estimated worldwide incidence of the HM and chorioncarcinoma is ~1 and 0.02/0.07 per 1,000, respectively (2, 6).

In the pre-chemotherapy era, invasive mole mortality was ~15%, caused by hemorrhage, sepsis, embolism, or surgical complications, whereas choriocarcinoma mortality was ~100% and ~60% in case of metastatic and non-metastatic disease, respectively (7, 8).

Currently, the cure rate is 90%. The risk of further molar pregnancy as well as chemotherapy-related fertility problems are the main issues (7–13).

Aim of the present study was to assess the incidence, prognosis, and obstetric outcomes of GTD patients admitted at an Italian university hospital. Furthermore, it was assessed the relationship between serum hCG levels and early identification of patients at risk of disease persistence.

## Materials and Methods

GTD cases were retrospectively reviewed from 2000 to 2020. Patients were retrieved using the report of histological examinations performed on surgical specimens and that were analyzed in the Institute of Pathology of the University of Sassari.

Each patient was then evaluated through a critical collection of the anamnestic, clinical and epidemiological information reported in their medical records.

This analysis was not reviewed by the local Ethics Committee of University of Sassari, Italy, because it was a retrospective study.

Demographic, epidemiological, and clinical characteristics were collected, including its the persistence and resistance to chemotherapy.

Serum hCG levels were measured after 1, 2, 3, and 4 weeks after uterine vacuum aspiration by Karman’s cannula (suction curettage) to predict the risk of GTD persistence.

All patients underwent a weekly follow-up based on the assessment of serum hCG levels, a gynecological examination, and ultrasound. The clinical evaluation was interrupted after two to three consecutive negative serum hCG levels.

We evaluated risk factors associated with the persistence of disease such as, serum hCG levels rise (or not reduction), maternal age >40 years and volume of endocavitary material.

An *ad hoc* electronic form was used to collect demographic, epidemiological, and clinical variables. Qualitative variables were described with absolute and relative (percentage) frequencies. Quantitative variables were summarized with means (standard deviations, SD) and medians (interquartile ranges, IQR) in case of normal and non-normal distribution, respectively. Individuals with and without persistent pathology were compared: chi-squared or Fisher exact test was used for the qualitative variables, Student t or Mann-Whitney test was used for normal and non-normal quantitative variables, respectively. ROC curve was used to assess the accuracy of serum hCG levels (1, 2, 3 weeks, and one month) in the prediction of the persistence of trophoblastic disease.

Sidak’s adjustment was carried out for multiple comparisons.

## Results

### Study Population and Diagnosis

A total of 54 patients were reported in the study period (2000–2020): forty-six (85.18%) were HM and 8 (14.81%) GTN (6, 75%, with a non-metastatic disease). Three HM were randomly diagnosed after histological examination on specimen recovered during suction curettage performed in 1 case for miscarriage and in 2 cases for voluntary termination of pregnancy. Two out of 8 cases (25%) GTN were metastatic. In both cases, the metastases occurred in the lungs, in particular in one case the radiological image found was the characteristic “*snow storm*” picture.

Three out of 8 (37.5%) GTN cases were choriocarcinoma. In one case the diagnosis was made on surgical specimen recovered by suction curettage carried out two months after spontaneous delivery; another case was found to be a primitive tubal choriocarcinoma after laparotomic salpingectomy performed urgently for acute hemoperitoneum; the third case was diagnosed with certainty only after the hysterectomy performed after chemotherapy with methotrexate (MTX) and folinate calcium (FC).

In most of the cases (53), the diagnosis was performed using the surgical specimen collected through uterine vacuum aspiration by Karman’s cannula (suction curettage).

Twenty-four (44.44%) patients, including those with a histopathological diagnosis of GTN, showed steady or slightly increase of serum hCG levels. Then, (after chest XR and total body CT to check for distant metastases), they were exposed to a first-line chemotherapy with MTX and FC; unfortunately, 6 (25%) were resistant and were treated with second-line drugs EMA/CO (etoposide, methotrexate, actinomicina-D, ciclophosphamide, and vincristine).

Six out of 24 (25%) patients undergoing chemotherapy, received total hysterectomy in order to report serum hCG levels to normal in three consecutive draws at the end of chemotherapy treatment, because being women over 40 years and not desiring pregnancy.

### Incidence of GTD

The incidence of GTD was 1.8 cases per 1,000 deliveries and 1.3 cases per 1,000 pregnancies (**Table 1**); in particular, the incidence of HM was 1.6 cases per 1,000 deliveries and 1.2 cases per 1,000 pregnancies. The incidence of GTN was 0.3 cases per 1,000 deliveries and 0.2 cases per 1,000 pregnancies.

**Table 1:** Incidence of gestational trophoblastic disease (GTD) x 100000 deliveries and x 100000 pregnancies.

Year	Incidence HM/deliveries	Incidence HM/pregnancies	Incidence GTN/deliveries	Incidence GTN/pregnancies	GTD/deliveries	GTD/pregnancies
2000	135.59	94.25	0.00	0.00	135.59	94.25
2001	260.76	182.98	65,19	45,74	325.95	228.73
2002	127.71	89.69	63.86	44.84	191.57	134.53
2003	180.94	131.93	0.00	0.00	180.94	131.93
2004	0.00	0.00	60.31	43.71	60.31	43.71
2005	316.46	224.92	0.00	0.00	316.46	224.92
2006						
2007	127.71	91.19	127.71	91.19	255.43	182.40
2008	64.39	45.21	0.00	0.00	64.39	45.21
2009	191.08	139.02	0.00	0.00	191.08	139.02
2010	125.39	92.38	0.00	0.00	125.39	92.38
2011						
2012	76.28	52.86	76.28	52.86	152.56	105.71
2013	236.78	180.07	0.00	0.00	236.78	180.07
2014	412.54	305.81	0.00	0.00	412.54	305.81
2015	78.37	58.17	0.00	0.00	78.37	58.17
2016	86.88	62.00	86.88	62.00	173.76	123.99
2017	265.49	187.27	0.00	0.00	265.49	187.27
2018	354.30	255.92	0.00	0.00	354.30	255.92
2019	69.93	50.35	0.00	0.00	69.93	50.35
2020	303.03	210.38	101.01	70.13	404.04	280.50
**2000**–**2020**	**155.37**	**111.63**	**27,02**	**19.41**	**182.39**	**131.05**

HM, hydatidiform mole; GTN, gestational trophoblastic neoplasia; GTD, gestational trophoblastic disease. Bold values: overall incidence of GTD in the last 20 years.

Among GTN, incidence of choriocarcinoma was: 0,1 cases/1000 deliveries and 0,07 cases/1000 pregnancies.

The highest prevalence was found in patients aged 31 to 40 years (33.3%) and 41–50 (31.4%). 50% were multiparous, with 16 (29.6%) with ≥1 miscarriage. The diagnosis was performed between the 7^th^ and 11^th^ weeks of gestational age. One patient had a previous history of GTD (3 molar pregnancies in 1988, 1991 and 1993). A patient developed a vesicular mole during an ovarian stimulation treatment for medically assisted reproduction technologies.

### Sign and Symptoms, Serum hCG Levels, Follow-Up, and Therapy

72% complained symptoms at diagnosis: 87% with vaginal bleeding and 51% with an increased uterine volume.

Almost half of the cases (25, 46.3%) showed the “*snow storm*” ultrasound finding.

Thirty (55.6%) patients had complete regression of serum hCG levels to normal without chemotherapy; 24 (44.4%) patients with a persistent trophoblastic disease were treated (**Table 2**). 18 (75%) patients had a complete remission after a mean of 3 cycles.

**Table 2:** Patients treated by first-line (MTX/CF) and second-line (EMA/CO) chemotherapy. CT).

Patient	Histology	CT (MTX/CF)	Response to therapy	EMA/CO	Hysterectomy (Hy) and bilateral anesectomy (Ba)
1	HM	3 cycles	Complete	–	–
2	HM	2 cycles	Complete	–	–
3	HM	2 cycles	Complete	–	–
4	Chorioncarcinoma	3 cycles	Resistance	8 cycles with CR	–
5	GTN	3 cycles	Resistance	6 cycles with CR	–
6	HM	3 cycles	Complete	–	–
7	HM	2 cycles	Complete	–	–
8	HM	2 cycles	Complete	–	–
9	GTN	3 cycles	Resistance	4 cycles con CR	–
10	HM	3 cycles	Complete	–	–
11	HM	3 cycles	Complete	–	–
12	GTN	8 cycles	Resistance	3 cycles with CR	–
13	GTN	3 cycles	Resistance	4 cycles with CR	–
14	HM	2 cycles	Complete	–	Hy+Ba after CT
15	HM	4 cycles	Complete	–	–
16	GTN	3 cycles	Resistance	2 cycles with CR	Hy+Ba after CT
17	HM	2 cycles	Complete	–	–
18	HM	4 cycles	Complete	–	Hy+Ba after CT
19	HM	4 cycles	Complete	–	Hy+Ba after CT
20	HM	3 cycles	Complete	–	Hy+Ba after CT
21	Chorioncarcinoma	7 cycles	Complete	–	Hy+ Ba during CT
22	HM	4 cycles	Complete	–	–
23	HM	3 cycles	Complete	–	Hy+Ba after CT for recurrent metrorragies
24	Primitive tubal chorioncarcinoma	5 cycles	Complete	–	–

HM, hydatidiform mole; CR, complete remission; GTN, gestational trophoblastic neoplasia.

Seven (29.2%) patients underwent laparoscopic or laparotomic hysterectomy at the end of the chemotherapy cycles and after three negative serum hCG levels. The surgically treated patients had an average age of 43 years (with a range of 41–51), at least two children and did not want to preserve fertility. The surgical treatment was carried out at the end of chemotherapy therapy, after regression of serum hCG levels to normal in three consecutive draws.

Of the 24 patients undergoing chemotherapy: 7/24 (29,17%) underwent hysterectomy with a mean age of 43 years, multiparous and without further desire for pregnancies; 8/24 (33,33%) were between the ages of 40 and 50 and had at least one child.

### Serum hCG Monitoring and Risk Factors for Disease Persistence

Serum hCG levels >100,000 mUI/ml at diagnosis increased the risk of disease persistence (OR: 10; p-value: 0.001).

Serum hCG levels >10,000 mUI/ml 1 week after suction curettage increased the risk of persistence (OR: 8.6; p-value: 0.007). Furthermore, a 1-month serum hCG levels between 100 and 1,000 mUI/ml, as well as at two-month between 10 and 100 mUI/ml, significantly increases the risk (OR 52.2; p-value: 0.001; OR: 9; p-value: 0.004, respectively). The risk can be increased by a positivity at three months (OR: 16; p-value <0.001). The ultrasound finding of the “*snow storm*” at diagnosis similarly increased the risk of disease persistence (OR: 3.3; p-value: 0.04).

**Figure 1:**
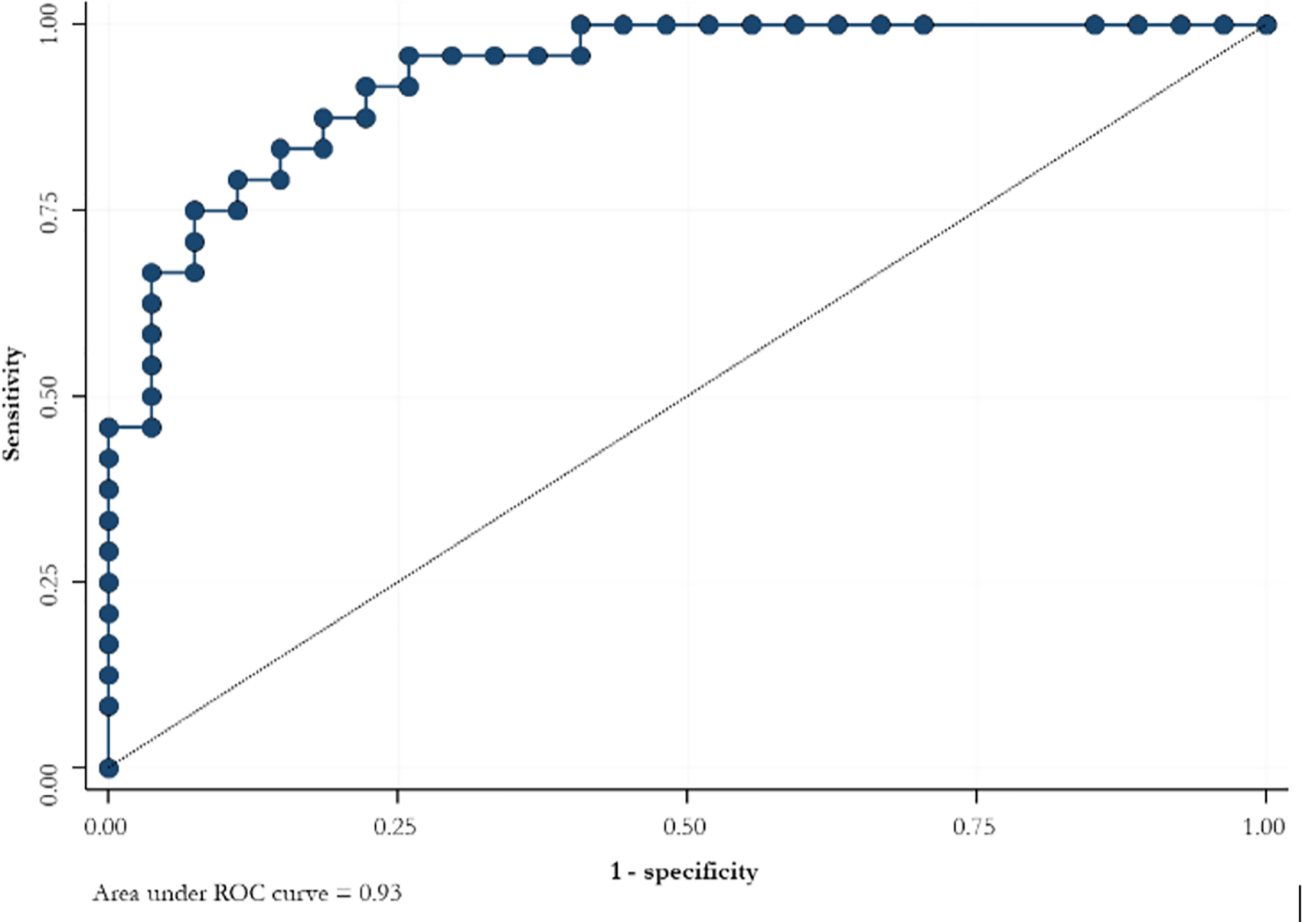
ROC curve for the slope of serum hCG levels at 3 weeks after suction curettage to predict the persistence of trophoblastic disease.

ROC curve for serum hCG levels at 3 weeks after the suction curettage predicted the persistence of trophoblastic disease with a sensitivity of 70.8% and a specificity of 92.6% (**Figure 1**).

Serum hCG levels between 100 and 1,000 mUI/ml at 1 month after suction curettage increased the risk of resistance to chemotherapy (OR: 14.1; p-value: 0.03). Similarly, a 2-month serum hCG levels between 10 to 100 mUI/ml and >100 mUI/ml increased the risk by 8 and 17 times, respectively.

Three month serum hCG levels positivity increased the risk of resistance to first-line chemotherapy by 12 times (OR 12.3; p-value: 0.005).

Serum hCG levels >100,000 mUI/ml were risk factors for the development of a trophoblastic disease. Maternal age >40 years and increased volume of endocavitary material increased the risk of disease persistence without any statistical significance.

### GTD and Obstetric Outcome

Nine out of 24 patients who had chemotherapy, afterwards 6 (66.67%) had at least one pregnancy following chemotherapy treatment. There were 10 pregnancies in total, including: 6 term deliveries without obstetric complications and no newborns presented chromosomal pathologies; 1 miscarriage at the first trimester; 2 interruptions of pregnancy; 1 extrauterine pregnancy.

Thus, 6 (66. 7%) patients had ≥ 1 pregnancy following chemotherapy. The survival of the cohort was 100%.

## Discussion

The incidence of GTD in our university hospital was 1.8 cases per 1,000 deliveries and 1.3 cases per 1,000 pregnancies during the time period 2000 to 2020. It is higher if compared with the Italian incidence (0.7–0.8 cases per 1,000 deliveries and 0.5 cases per 1,000 pregnancies) (13–21). The increased epidemiological burden of GTD could be explained by unknown genetic factors, selected in Sardinia island such as autoimmune diseases (e.g., diabetes mellitus type 1, multiple sclerosis, and celiac disease) whose prevalence and incidence are highest. This hypothesis is supported by a study carried out in indigenous villages in Alaska, where the incidence of the hydatidiform mola was 3.9 cases per 1,000 deliveries (22). Mutations of the NLRP7 gene on chromosome 19q were found in families with recurrent vesicular mole (23). This gene involved also in mediating inflammatory pathways may represent a milestone in linking GTD with autoimmune diseases, further investigations will be attempted in our Institution to clarify the role of this genetic pattern in Sardinian women.

The incidence of choriocarcinoma was 0.072 cases per 1,000 pregnancies and was higher than that estimated in Europe and USA (0.02 cases per 1,000 pregnancies), whereas it was comparable to that of Japan (0.075 cases per 1,000 pregnancies) (23, 24).

It was found a reduction of GTD incidence when the current analysis was compared with a previous one performed for a cohort recruited between 1976 and 1989 (1.8 per 1,000 deliveries VS. 3.6 per 1000 deliveries, respectively) but a slight increase if compared with the incidence of the cohort enrolled between 1974 and 1983 (1.46 per 1,000 deliveries). On the other hand, the incidence of choriocarcinoma was almost comparable (0.04 per 1,000 deliveries of 1974–1983 VS. 0.06 per 1,000 deliveries of 1976–1989 VS. 0.07 per 1,000 deliveries of 2000–2020) (12, 13).

The results on the factors associated with the persistence of the disease are in agreement with previous findings; serum hCG levels >100,000 mUI/ml are a risk factor for the development of a trophoblastic disease (25–28). Other risk factors associated with the persistence of disease are maternal age >40 years and increased volume of endocavitary material; in our cohort both factors increased the risk of disease persistence without any statistical significance.

Serum hCG levels monitoring is mandatory in the follow-up of patients with molar pathology to perform an early diagnosis of persistent trophoblastic disease and to diagnose patients with resistance to first-line chemotherapy (9, 29–31).

It would be helpful to identify a serum hCG levels threshold: the analysis carried out in our cohort found a good specificity and a limited sensitivity. A large sample size could increase the accuracy. The identification of an appropriate threshold of hCG serum levels in monitoring GTD is of great relevance, also considering the recent findings of a large meta-analysis which documented a very favorable obstetric outcome in women receiving conservative management of complete/partial molar pregnancy (32), thus highlighting the need to properly follow up women with GTD.

We acknowledge that the retrospective nature, and the long period of patients’ enrollment represent major study limitations; however, the homogeneity of investigated population is certainly a relevant strength.

## Conclusions

Although GTD is a rare disease, its incidence was 1.3 cases per 1,000 pregnancies in Sardinia, Italy, higher if compared with mean national and worldwide incidence. Genetic factors could concur to the increased burden.

## Data Availability Statement

The original contributions presented in the study are included in the article/**Supplementary Material**. Further inquiries can be directed to the corresponding author.

## Ethics Statement

Ethical review and approval was not required for the study on human participants in accordance with the local legislation and institutional requirements. Written informed consent for participation was not required for this study in accordance with the national legislation and the institutional requirements.

## Author Contributions

Project development: GC and ET. Data collection and manuscript writing/editing: LS and FD. Data collection and manuscript editing: MP and MM. Project development, data management, and manuscript editing: GV, AO, and DS. Manuscript writing/editing: AC, SD, and PC. Data analysis: GS. All authors contributed to the article and approved the submitted version.

## Conflict of Interest

The authors declare that the research was conducted in the absence of any commercial or financial relationships that could be construed as a potential conflict of interest.
